# Implementing MIA – Mannheim's interprofessional training ward: first evaluation results

**DOI:** 10.3205/zma001243

**Published:** 2019-08-15

**Authors:** Mira Mette, Christina Baur, Jutta Hinrichs, Elke Oestreicher-Krebs, Elisabeth Narciß

**Affiliations:** 1 Heidelberg University, Medical Faculty Mannheim, Division for Study and Teaching Development, Mannheim, Germany; 2 Heidelberg University, University Hospital Mannheim, Medical Faculty Mannheim, Department of Medicine II, Mannheim, Germany; 3 Academy of University Medical Centre Mannheim, School of Physiotherapy, Mannheim, Germany; 4 Academy of University Medical Centre Mannheim, School of Nursing, Mannheim, Germany

**Keywords:** interprofessional learning, interprofessional teaching, training ward, health care professions, education

## Abstract

**Project description:** In Germany there is great interest in better preparing learners in the health care professions for interprofessional (IP) collaboration on IP training wards. On the MIA, Mannheim’s interprofessional training ward, medical students, nursing apprentices and physiotherapy (PT) trainees learn and practise real patient care in a team under supervision. The concept of the MIA, its implementation and the first evaluation results are reported. During the 2017/18 academic year, 201 medical students, 72 nursing apprentices and 33 PT trainees completed their mandatory placements on the MIA, which they evaluated online at the end of the placement (questions on the organisation of the MIA placement, learning gains, supervision, participant satisfaction, personal insights). The data was analysed according to frequency for each health care profession separately using the Kruskal-Wallis test for comparing the evaluation data between the three participant groups.

**Results: **The response rate was 45% (104 medical students, 16 nursing apprentices, 19 PT trainees). 64% of the medical students considered the placement too short. For 70% of the nursing apprentices, the number of patients to be treated was too high. The supervision by the facilitators was adequate. There were often IP contacts. Professional and IP learning gains were rated high. IP learning took place mainly in personal conversations and on IP ward rounds. IP communication/collaboration was mentioned most often as an important insight gained from the placement.

**Discussion: **The implementation of the MIA concept is considered successful. The learning objectives were achieved. The structured daily routine on the ward with its IP elements promotes IP collaboration and helps to minimise difficulties in the clinical placement, which – often for the first time – demands that the participants manage patient care in an accountable manner.

**Conclusion:** Placements on IP training wards in the education of health care professionals can be a good preparation for practising optimal patient care in the future.

## Zusammenfassung

**Projektbeschreibung: **In Deutschland ist das Interesse groß, Lernende in den Gesundheitsberufen auf interprofessionellen (IP) Ausbildungsstationen besser auf die IP Zusammenarbeit vorzubereiten. Auf der Mannheimer IP Ausbildungsstation MIA lernen und üben Medizinstudierende, Pflegeauszubildende (Pflegeazubis) und PhysiotherapieschülerInnen (PT-SchülerInnen) die reale Patientenversorgung im Team unter Supervision. Das MIA-Konzept, die Umsetzung und erste Evaluationsergebnisse werden berichtet. Im Studienjahr 2017/18 absolvierten 201 Medizinstudierende, 72 Pflegeazubis und 33 PT-SchülerInnen ihren Pflicht-Einsatz auf der MIA, den sie am Ende online bewerteten (Fragen zu Organisation des MIA-Einsatzes, Lernzuwachs, Supervision, Teilnehmerzufriedenheit, Erkenntnisse). Die Auswertung erfolgte berufsgruppenspezifisch nach Häufigkeiten und im Vergleich per Kruskal-Wallis-Test.

**Ergebnisse:** Die Rücklaufquote lag bei 45% (104 Medizinstudierende, 16 Pflegeazubis, 19 PT-SchülerInnen. 64% der Medizinstudierenden fanden den Einsatz zu kurz. Für 70% der Pflegeazubis war die Zahl der zu versorgenden PatientInnen zu hoch. Die Betreuung durch SupervisorInnen war angemessen. Es gab häufig IP Kontakte. Fachlicher und IP Lernzuwachs wurden hoch eingeschätzt. IP Lernen fand vor allem in persönlichen Gesprächen und IP Visiten statt. Am häufigsten wurde IP Kommunikation/Zusammenarbeit als wichtige Erkenntnis des Einsatzes genannt.

**Diskussion: **Die Umsetzung des MIA-Konzepts wird als gelungen beurteilt. Die Lernziele konnten erreicht werden. Der strukturierte Stationsalltag mit seinen IP Elementen fördert die IP Zusammenarbeit und hilft, Schwierigkeiten im teils ersten eigenverantwortlichen Klinikeinsatz zu minimieren.

**Schlussfolgerung:** IP Ausbildungsstationen können in den Ausbildungen der Gesundheitsberufe eine gute Vorbereitung für eine spätere optimale Patientenversorgung bieten.

## Introduction

Internationally seen, Germany only began reducing the backwardness in interprofessional (IP) education of health care professionals in 2013, with support from the Robert Bosch Stiftung's “Operation Team” funding program [1], [2]. Initially, individual and highly differing learning scenarios were developed ranging from lectures, seminars, practical sessions and workshops to simulations [2], [3]. Since 2014 different IP learning sessions have been developed at the University Medical Centre Mannheim and interlinked to a longitudinal IP learning curriculum sequence [4]. After visiting different IP training wards in Stockholm, Sweden, it was decided to complete the curriculum sequence by developing and implementing MIA, Mannheim’s IP training ward, according to the Swedish model. Sweden, along with Denmark, is a pioneer in establishing IP training wards as an integral part of the education of health care professionals. These countries have more than 20 years of experience in how students of medicine, nursing, physiotherapy (PT) and occupational therapy can plan, practise and reflect on the care of real patients in a team under supervision and do this in an accountable manner [5], [6], [7], [8], [9]. Supervision is a way of facilitating the learning processes on the IP training ward. It involves the profession-specific supervision of the learners of one's own health care profession as well as the learners of all professions regarded as a team that needs guidance in collaboration and reflection [5], [8]. In addition to working together in patient care, future professionals learn to apply theoretical knowledge and practical skills in a real-life context, to take on responsibility, to understand their own professional role and that of other professions, and to work together in a team for the benefit of patients [5], [8], [10], [11], [12].

This project report aims to answer the question of whether it is also possible in Germany to implement an IP training ward with mandatory placements for the three professions medicine, nursing and PT in a way that gives all participants a positive impetus in the development of their own professional role in the team and the understanding of the competences of the other professions.

## Project description

In 2017 the usual practical course in internal medicine in the fifth year of medical study at the Medical Faculty Mannheim, Germany, was transformed into a clinical placement on an IP training ward (cf. [5], [8], [13], [14]). The concept envisages that medical students, nursing apprentices and PT trainees are trained together for a period of one to three weeks, thus learning and practicing collaboration in the IP team in everyday clinical practice. Various institutions were significantly involved in developing the concept of the MIA (see table 1 (Tab. 1)).

### Framework of the MIA

The MIA was defined as half of a gastrointestinal and infectious diseases ward (12 beds). A preselection of patients does not take place; however, the patients are informed about the special concept of the MIA upon admission. On the MIA, fifth-year medical students work together with nursing apprentices (mainly third year of apprenticeship) and PT trainees (mainly second year of training). They take on patient care in a team under supervision. MIA placements are mandatory for all learners. The precondition of operating the MIA all year round and the general restrictions of the three health care education programmes require that the duration of the placement varies: medical students complete one week, PT trainees two weeks and nursing apprentices three weeks. During the semester break, medical students in their final year (sixth year of study) take over and work together with the nursing apprentices and PT trainees on the MIA whose placements are planned all year round. Due to PT education restrictions, the MIA runs for ten weeks spread out over the year with only nursing apprentices and medical students in their final year.

The professional preparation of the learners for their MIA placements takes place monoprofessionally, e.g. through seminars or on-site briefings. All learners get information about the MIA placement, e.g. organisational matters, learning objectives, evaluation criteria, etc. from their educational institution or online via Moodle. The fifth-year medical students complete the placement with graded practical examinations as part of the overall certificate of completion for the “Practical Course in Internal Medicine”. The PT trainees and nursing apprentices complete part of their clinical training in internal medicine on the MIA. Their performance is evaluated by the responsible facilitators. Due to their high numbers, fifth-year medical students (approximately 200 per year) work in short alternating shifts of six hours each, while the nursing apprentices take over regular early shifts and late shifts. Due to course scheduling, PT trainees only work in the mornings. Three health care professions work on the MIA in the morning and two in the afternoon. Night shifts are done by the regular staff. On public holidays and weekends there is no IP patient care on the MIA, as only nursing apprentices are on duty. A shift consists of 4-6 medical students, 2-3 nursing apprentices and 1-2 PT trainees who care for 12 patients under the supervision of experienced, specially trained facilitators from each profession. The IP team has its own meeting room or office which is equipped with sufficient PC workstations. For the MIA placement there are global IP learning objectives (see table 2 (Tab. 2)) as well as professional, profession-specific learning objectives.

#### IP patient care on the MIA

**Figure 1:**
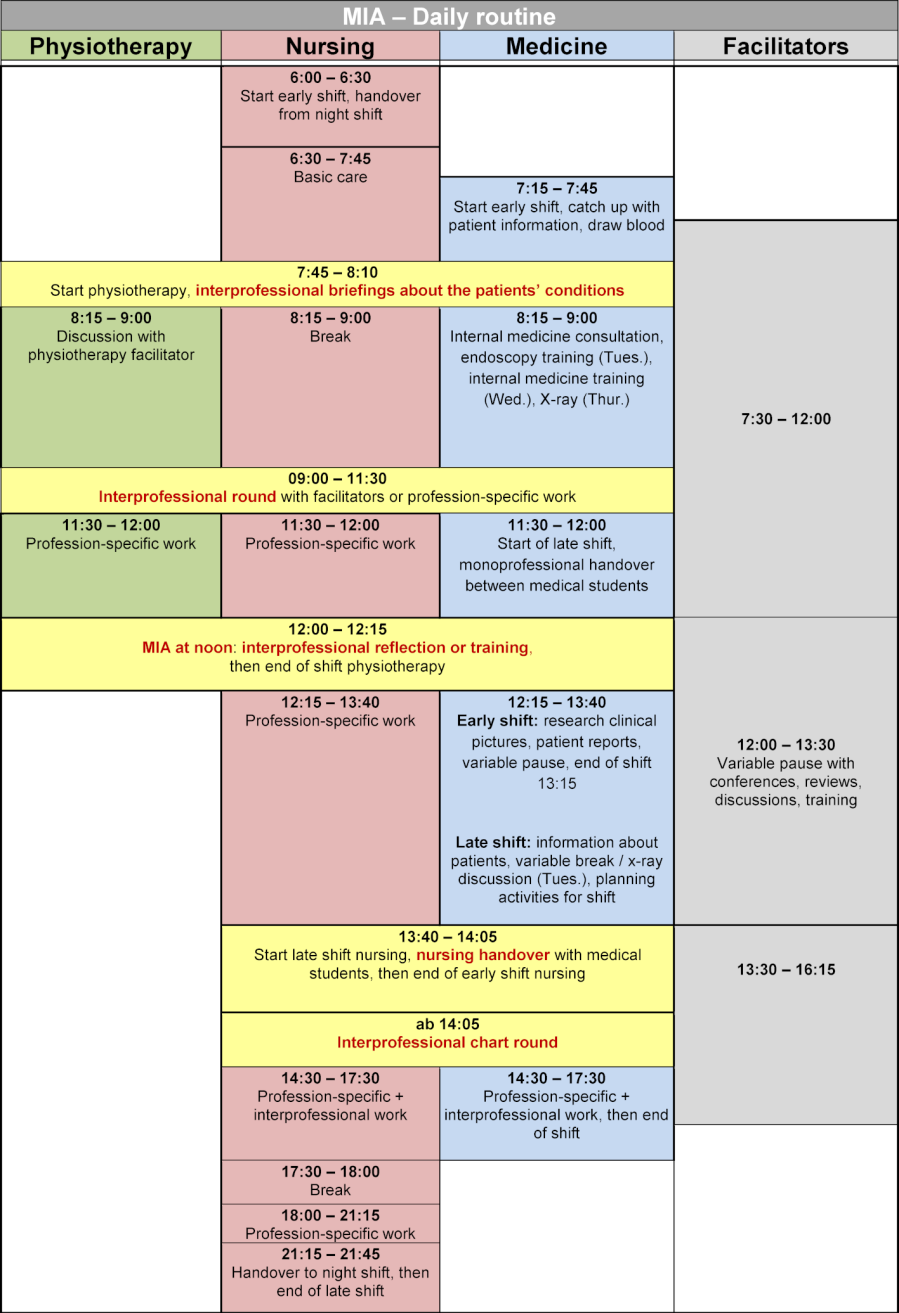
Daily routine on the MIA.

The daily routine on the MIA provides the following fixed time slots for IP exchange (see figure 1 (Fig. 1)):

1. IP briefing about all patients’ conditions including identifying and prioritising physiotherapy need
2. ward round with all learners of all three professions responsible for the respective patients, including the facilitators
3. MIA at noon: reflection time for getting to know each other, sharing expectations, fears, experiences (weekly conclusion); short IP peer-teaching sessions on relevant skills
4. handover nursing followed by chart round

#### Supervision on the MIA

The facilitators are experienced senior and assistant doctors, nursing instructors and PT teachers. The core idea of the MIA concept is that the facilitators do not instruct in the traditional way, rather they step into and stay in the background. From there they observe the (inter-)actions of the IP team of learners in providing patient care and during the meetings. The learners are given guidance to communicate in order to plan and implement patient care together. This learner-centred and activating approach should help to encourage the learners to take on individual responsibility in patient care. The future health care professionals and not the facilitators should be the contact persons for the patients. However, the facilitators must be able to intervene at any time to ensure patient safety. It is also their task to evaluate the performance of “their” learners. The facilitators were trained for this special form of “teaching” in a one-day workshop that was held by a former nursing facilitator and leader of facilitator teams with many years of experience on Swedish IP training wards.

#### Evaluation of the MIA placements

The MIA placements are evaluated voluntarily and anonymously by the participants as part of the regular course evaluation. Validated questionnaires in German such as RIPLS [15], UWE-IP [16], IEPS [17], which focus on attitudes or changes in attitudes towards IP learning, are not suitable for evaluating the implementation of the new teaching and learning method. Thus, an online questionnaire with 27 open-ended and closed questions (3-point or 5-point Likert items) was developed which the MIA participants can access via a TAN on the last day of the placement. Following some general questions, e.g. on the profession, the level of education and the prior interest in IP learning, the participants assess the organisation and preparation for the MIA placement, the learning gains and the supervision. They give an overall assessment of their placement and name important insights gained during the placement (see table 3 (Tab. 3)). 

Within 35 weeks of the 2017/2018 academic year, 210 medical students (9 of them in their final year who are not considered the main target group of medical students and are therefore not included in the results), 72 nursing apprentices and 33 PT trainees completed their MIA placements. The analysis of the survey data was based on frequency; the Kruskal-Wallis test with independent samples was used for comparison of the three professions. A quantitative content analysis with inductive category formation was carried out for the open-ended questions.

The aim of the formative evaluation was to find out through the assessment of the MIA participants how well the MIA concept was implemented and how satisfied the participants were with the new teaching and learning method. Moreover, aspects to be optimised could be identified in order to improve the quality of IP education on the MIA.

## Results

The MIA started with the beginning of the 2017/18 winter semester in the fifth year of medical study. A total of 139 MIA participants participated in the survey. The response rate (45%) varied according to the profession (see table 4 (Tab. 4)).

The retrospective view on the interest in IP learning prior to the placement on the MIA was high (medicine: 87%, nursing: 81%, PT: 79%). While most of the nursing apprentices and PT trainees considered the duration of the MIA placement to be appropriate (nursing: 57%, PT: 79%), the majority of the medical students (64%) found their one-week placement too short. Medical students (99%) were responsible for 2-3 patients per day on average, nursing apprentices (94%) for more than 4 patients and PT trainees (90%) for 2-3 patients. Medical students (86%) and PT trainees (95%) considered the number of patients to be adequate. Many nursing apprentices (70%) found the number of patients assigned to them too high.

The evaluation results of the participants’ self-reported learning gains are shown in table 5 (Tab. 5). No significant differences between the professions were found for any of the items.

The amount of personal supervision provided by facilitators was judged by the majority to be appropriate (medicine: 82%, nursing: 60%, PT: 95%). The same applied to the level of acting in an independent and accountable manner on the MIA (medicine: 86%, nursing: 70%, PT: 95%).

**Figure 2:**
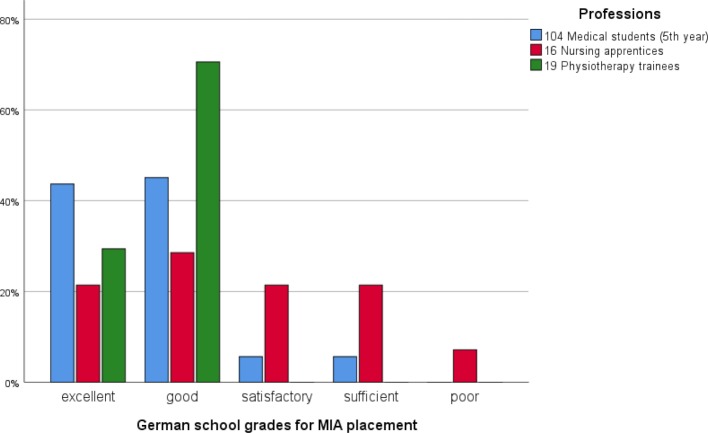
Overall evaluation of the MIA placement by profession using the conventional German grading scale.

Almost all participants agreed that there was frequent contact with the other professions (medicine: 83%, nursing: 94%, PT: 90%) and that the IP collaboration during the placement was very good (medicine: 98%, nursing: 100%, PT: 100%). The overall assessment of the MIA placement, based on the German school grading system (1=very good, 5=poor), was mostly very positive (see figure 2 (Fig. 2)).

The free-text answers (multiple answers possible) showed that personal conversations and ward rounds were most often stated as opportunities to learn about the other professions. Among the three most important insights mentioned from the MIA placement were IP communication and collaboration, self-organisation and the prioritising of tasks, as well as gaining knowledge of the organisation and procedures of the ward (see table 6 (Tab. 6)).

## Discussion

The results of the online survey show that the implementation of the new IP teaching method in the clinical context with its independent and accountable work and personal supervision by the facilitators is approved of by the participants. Above all, they see an additional benefit in experiencing IP communication and collaboration in the joint patient care which is in line with the results of international studies [5], [10], [12]. This confirms the successful implementation of the new teaching method. The high interest of the participants in learning together with other professions in a real clinical context prior to their MIA placement is regarded as a good basis for successful MIA placements. 

However, IP learning on the ward places high demands on all professions. On the MIA, medical students usually have the first opportunity to take on medical responsibility and to practise real patient care. The intensive supervision provided by the facilitators is highly appreciated – possible fears of making mistakes or not living up to expectations can be compensated for in this way. The medical students’ opinion on their one-week placement being too short may be due to the fact that, prior to this placement, most medical students are not used to providing comprehensive care to patients assigned to them while actively mastering the daily ward routine with all the associated activities. Once they are familiar with the IP ward work, the placement is already finished. An extension of the placement would be desirable in order to enhance the clinical collaboration in the team and to achieve a more sustainable effect [8]. 

On the MIA, the nursing apprentices are expected to assume responsibility for nursing patients and running the ward to a degree that goes beyond the usual “assisting” tasks expected of them. Nursing apprentices are often excluded from collaborating with other therapy professions although it is they who are closest to the patients and thus know their situation and needs. On the MIA, the nursing apprentices seem to be more than fully occupied with the patients assigned to them, especially since they are often responsible for more than the intended four patients when their fellow apprentices are absent due to illness. Consequently, the nursing apprentices often considered the number of patients assigned to them too high.

During their practical training on the wards, PT trainees are often confronted with the situation that they have had hardly any opportunity to exchange information about the (current) condition of the patients before they see the patient. It is usually difficult to find out from the written prescription and the doctor's reports recorded in the electronic documentation which of the patient’s problems is crucial and therefore needs to be treated. The flow of information seems to work well through direct contact during the MIA placement. The MIA placement of PT trainees takes place early in the curriculum and as a result they often lack experience in dealing with multimorbid patients and/or professional competence. This can be compensated for by individual PT supervision, something that is rated very positively by the PT trainees. Moreover, IP meetings can help all professions to better recognise core areas of physiotherapy treatment and to focus more on patients’ level of activity, so that any further care and treatment by the team can build on this.

The structure of the daily routine on the MIA with fixed time slots for IP exchange seems to facilitate IP collaboration as hoped. Even if the daily routine is unusual at first, as it differs significantly from the usual routine of the individual profession – above all that of nursing –, everyone appreciates the formal exchange time slots with the other professions and perceives them as valuable. The frequent contact with the other professions confirmed by the participants leads to intensive communication and interaction between the learners. This allows them to learn on the MIA how important it is to collect and share relevant information with all professions [5], [10], [12]. IP learning situations arise, for example, when introducing patient cases at the IP briefing by the nursing apprentices, when medical students and PT trainees ask questions, or when the next treatment steps are discussed and prioritised. The success of the IP interactions is guided by the facilitators in the background as necessary. The participants also particularly appreciate the informal IP one-on-one conversations in which they learn a lot about the other professions. The shared meeting room or office contributes significantly to this.

The participants regard the IP ward round as the central IP element in which they learn about the other professions. At the same time, it allows the learners of the different professions who are responsible for the same patient to intensively exchange information about the patient and to act as a health care team. Supervised by the facilitators in the background, problems that arise during the pre-ward round discussion or during the round can be addressed, thus creating a safe learning environment. 

MIA at noon, the time slot for IP reflection and training for all participants, should allow a conscious pause from one's work for reflection on one's role in the team and that of the other professions. These reflection time slots were hardly mentioned as learning opportunities to learn about other professions [18]. Nevertheless, reflection is regarded as a core element of IP learning in order to develop good IP collaboration practice for the benefit of patients [19]. 

The high learning gains in the professional scope of practice and the different demands that are made on the respective profession in the daily ward routine – above all the ones made on the doctors – show that too little attention is paid to this topic in the three health care education programmes. However, knowledge about the other professions is the basis for good IP communication and collaboration [20], [21].

The combination of IP and professional learning seems to work on the MIA – both are important for the future work as a health care professional and should not be treated separately [9], [22]. Consciously experiencing IP communication and collaboration during the MIA placement can help to recognise that the quality of patient care can be optimised by working as an IP team [23]. This ideally manifests itself in IP collaboration in future professional life [5], [10], [12].

### Limitations

Among other things, it must be taken into account that the evaluation results are based on self-reported data. Due to the different duration and shift times of the placement of the professions involved, the new MIA teaching method is evaluated against different backgrounds in terms of educational level and clinical and IP experience. In addition, the response rate of the nursing apprentices is too low compared to the other professions. This may have been caused by the access via TAN to the online survey, which was new to the nursing apprentices.

## Conclusion

The teaching and learning concept of the MIA has been well implemented. The learning setting on a real hospital ward allows learning and becoming familiar with the organisation and the different procedures of one's profession and those of the other professions. The patients, who are often severely ill, demand that all learners communicate in the team, work together and make joint decisions. Even though the working hours and shifts of the professions do not presently match due to differences between the education programmes, the mandatory MIA placements bring students, apprentices and trainees of three professions together to take over joint patient care under supervision. The learners benefit from practising IP communication and collaboration on a real ward. 

The aim is to extend the MIA placement for medical students to two weeks. It would allow harmonisation of the placements with those of the PT trainees. This could strengthen team-building even more. 

Guaranteeing daily supervision by trained facilitators who continually ensure patient safety and the adequate implementation of the teaching and learning method is indispensable for running the MIA. In order to maintain a constant quality of supervision and improve the MIA, further workshops and meetings are regularly planned, in which new ideas are worked out and their possible implementation is discussed in addition to organisational matters.

## Acknowledgements

We would like to thank the numerous people involved in the conception and implementation of the MIA for their dedicated commitment. Special thanks go to Prof. Dr. Ebert and Priv.-Doz. Dr. Vogelmann of the Department of Medicine II and the Dean of Studies, Prof. Dr. Wieland, as well as Dr. Fritz-Joas, Head of the Study and Teaching Development Division. The supportive input from René Ballnus and his colleagues at the Karolinska Institute paved the way for implementing the MIA. The funding of the IP learning curriculum sequence by Robert Bosch Stiftung enabled us to think ahead and to extend IP learning to the real clinical context.

## Competing interests

The authors declare that they have no competing interests. 

## Figures and Tables

**Table 1:**
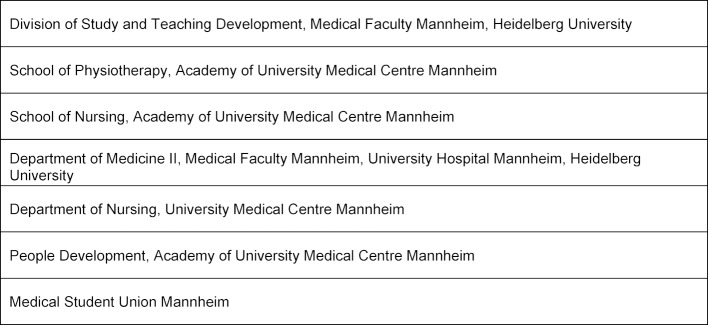
Institutions involved in developing the MIA concept

**Table 2:**
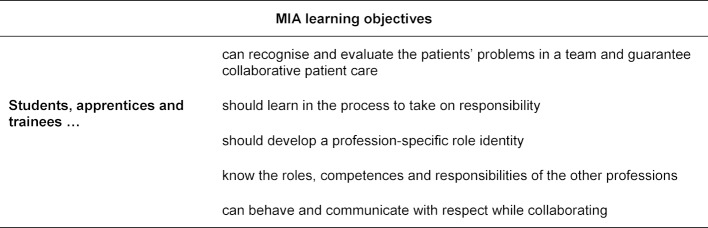
Interprofessional learning objectives of MIA

**Table 3:**
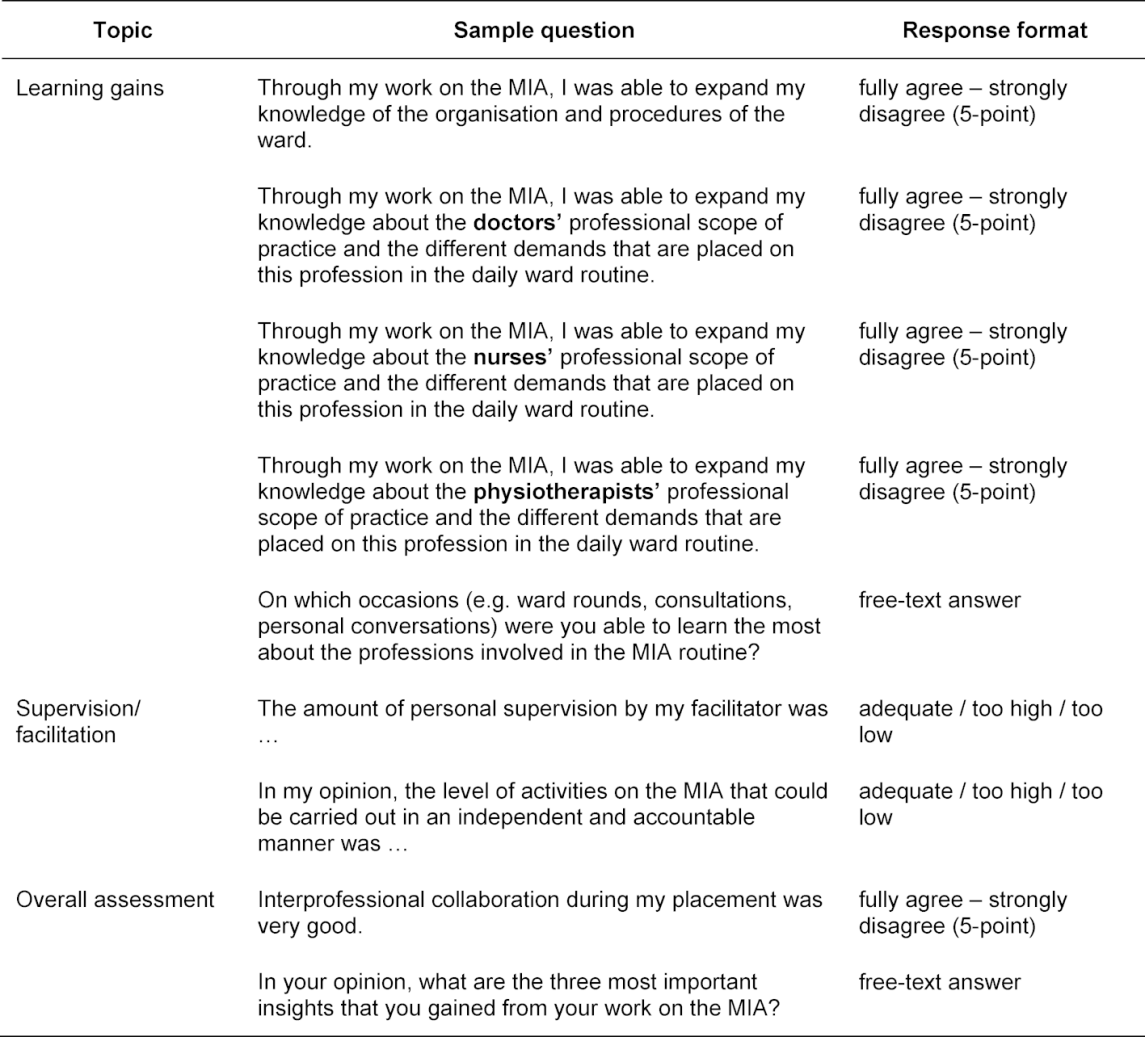
Sample questions from the online survey

**Table 4:**
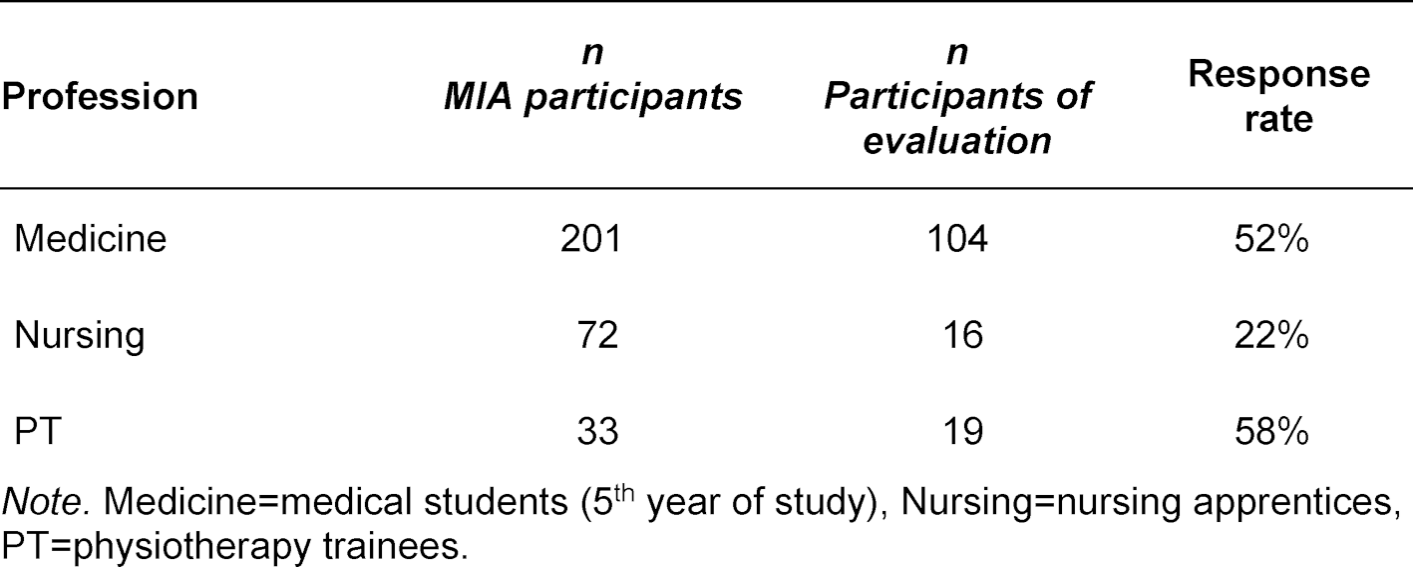
Composition of the participants by profession

**Table 5:**
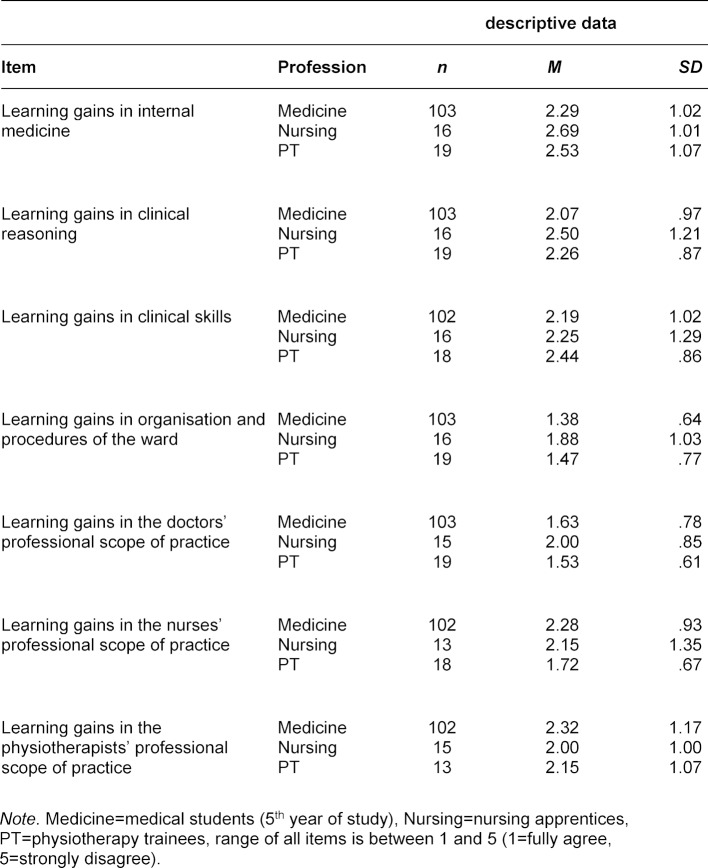
Evaluation results for learning gains during the MIA placement by profession

**Table 6:**
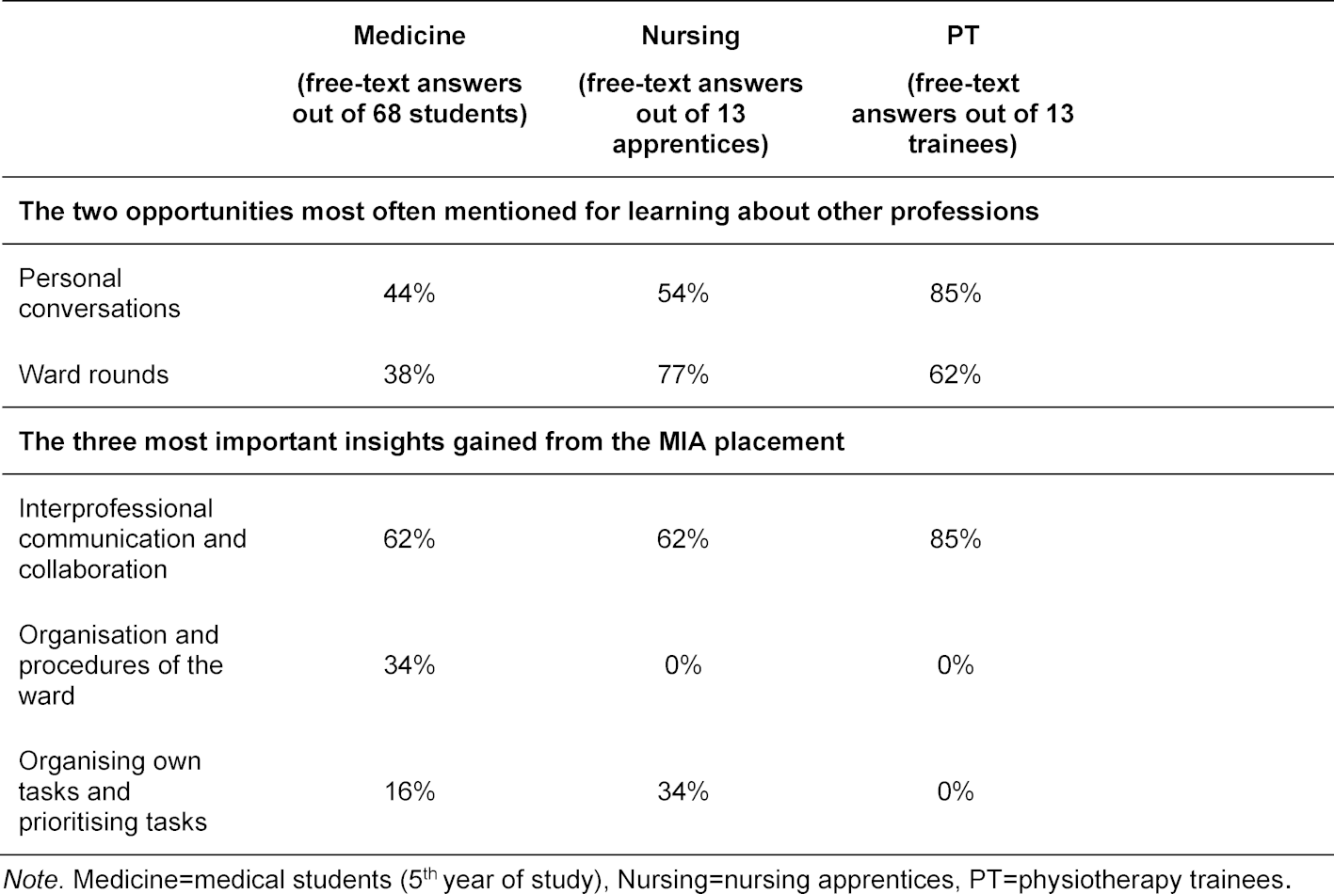
Evaluation results for free-text answers (multiple answers possible) by profession
